# The Effects of High-Intensity Functional Training on Cognition in Older Adults with Cognitive Impairment: A Systematic Review

**DOI:** 10.3390/healthcare10040670

**Published:** 2022-04-02

**Authors:** Yulieth Rivas-Campo, Patricia Alexandra García-Garro, Agustín Aibar-Almazán, Antonio Martínez-Amat, Gloria Cecilia Vega-Ávila, Diego Fernando Afanador-Restrepo, Felipe León-Morillas, Fidel Hita-Contreras

**Affiliations:** 1 Faculty of Human and Social Sciences, University of San Buenaventura—Cali, Santiago de Cali 760016, Colombia; yrivasc@usbcali.edu.co; 2 School by Faculty of Distance Learning and Virtual Education, Antonio José Camacho University Institution, Santiago de Cali 760016, Colombia; palexandragarcia@admon.uniajc.edu.co (P.A.G.-G.); gcvega@profesores.uniajc.edu.co (G.C.V.-Á.); 3 Department of Health Sciences, School of Health Sciences, University of Jaén, 23071 Jaén, Spain; amamat@ujaen.es (A.M.-A.); fhita@ujaen.es (F.H.-C.); 4 School for Faculty of Health Sciences, University Foundation of the Área Andina—Pereira, Pereira 660004, Colombia; dafanador4@areandina.edu.co; 5 Department of Physiotherapy, Catholic University of Murcia UCAM, 30107 Murcia, Spain; fleon@ucam.edu

**Keywords:** high-intensity functional exercise, older adults, general cognition, randomized controlled trials, cognitive impairment

## Abstract

(1) Background: High-Intensity Functional Training (HIFT) is a new exercise modality that emphasizes multi-joint functional movements adaptable to any fitness level and promotes greater muscle recruitment. Previous studies have evaluated the positive effects of HIFT on mental and cognitive health but have not evaluated it in older people. This study aims to conduct a systematic review of randomized controlled trials assessing the effects of HIFT on general cognition in older adults with cognitive impairment. (2) Methods: Following the PRISMA 2020 guideline, articles that did a high-intensity functional physical exercise intervention on cognitive performance in older adults with mild to moderate cognitive impairment (MMSE > 10) or dementia, aged 55 years or older, published between 2011 and 2021 in five different electronic databases: PubMed, Web of Science, Scopus, CINAHL, and Cochrane plus were included. (3) Results: 7 articles were included, all having general cognition as their primary outcome. All assessed general cognition using the Mini-Mental State Examination, the ADAS-Cog, or both. All studies had at least one HIFT experimental group with a frequency of 2 sessions per week and a variable duration between protocols of 12, 13, 16, and 26 weeks. Two articles showed that a progressive HIFT program improves general cognition, four articles showed no significant changes within or between groups and one article concluded that a HIFT intervention does not slow cognitive decline. (4) Conclusions: Evidence exists of the benefits of HIFT on general cognition in older adults with cognitive impairment, assessed using the MMSE, the ADAS-cog, or both. Two articles that showed improvement in cognitive function used progressive HIFT with 80% RM at 6, 12, and 1 weeks; however, in the other articles, due to the heterogeneity of intervention protocols, measurement time points, and control group activities, mixed results were evidenced

## 1. Introduction

Some of the most significant changes facing the world population are the increase in the number and proportion of older people [1] and the progression of life expectancy to older ages [2]. Cognitive deterioration is one possible consequence of the aging process, given that from the third decade of life the brain begins to atrophy, and its blood flow and weight decrease [3]. This greatly affects the functioning of the central nervous system [1], producing loss of memory, attention, reduced learning ability, and the deterioration of cognitive functions [4,5]. This decline is associated with an increased risk of dementia, as well as adverse health outcomes such as functional limitations and disability [6].

The burden on health systems caused by dementia and other adverse cognitive outcomes has become a major social challenge with great added financial costs [7]. New methods are required in order to prevent losses and even improve cognitive performance, functionality, autonomy, and quality of life in general [6]. For this reason, over the last decades interest has grown concerning the influence of lifestyle factors such as physical exercise on the prevention of cognitive impairment among older people [6].

Today, physical activity is deemed to be a highly protective factor of cognitive functions in normal brain aging, as well as in several stages of pathology-related cognitive deterioration [8,9]. Regular physical exercise has been associated with an increased brain volume of regions related to cognitive functions, which normally decline with age [10].

As of late, High-Intensity Interval Training (HIIT) has gained attention as a good choice of exercise for the young as well as the adult population. This type of exercise is characterized by brief and intermittent sessions of high-intensity activity alternating with periods of rest or low intensity. The number of studies that investigate this type of training in the older population has increased in recent years [2,11,12].

An alternative to HIIT is High-Intensity Functional Training (HIFT), a relatively new training modality that emphasizes multi-joint functional movements that can be adapted to any fitness level and lead to higher muscle recruitment than more traditional forms of exercise. HIFT sessions can last from two minutes to more than an hour [13]. It differs from HIIT in the use of constantly varied functional exercises and activities of adaptable duration that may or may not incorporate breaks [14]. HIFT employs multiple energy pathways through multimodal exercise utilization [15]. Because of the multiple prescription schemes related to exercise repetitions and durations in HIFT, programs can range from bodyweight exercises performed in circuits or timed intervals to more complicated schemes involving Olympic lifts, with a set number of repetitions [13].

Although HIIT AND HIFT share many similarities, they differ in that HIIT uses only aerobic exercises performed at very high intensity without variation [16], whereas HIFT uses constantly varied high-intensity functional and muscle-strengthening exercises of varying durations that may or may not incorporate breaks [14]. Similarly, studies suggest that HIFT is more effective than HIIT in increasing strength [17] and adherence to exercise [15,18], and strength training increases brain-derived neurotrophic factor [19] and IGF-1, [20] myokines important in cognition to a greater extent.

Over the last decade, studies evaluating the effectiveness of HIFT programs have documented improvements in metabolic [14] and cardiorespiratory adaptations [21], cognition [22,23], and overall health [2]. Numerous studies have shown the positive effects of HIFT programs on the mental and cognitive health of children [24], adolescents [22,25], and college students [26], and some systematic reviews have been published studying the effects of HIIT on cognition [27,28], however, there is none evaluating HIFT on cognition in older people. For that reason, this study aims to conduct a systematic review of randomized controlled trials to assess the effects of HIFT on cognitive abilities in older adults with cognitive impairment.

## 2. Materials and Methods

The bibliographic search and selection, data extraction, and systematic review were performed following the PRISMA 2020 guidelines [29]. The pre-specified protocol was that of the PROSPERO International Prospective Register of Systematic Reviews (CRD42022300929).

### 2.1. Eligibility Criteria

Articles were selected according to the following criteria: studies looking into the effects of a high-intensity functional physical exercise intervention on cognitive performance in older adults with mild to moderate cognitive impairment (MMSE > 10) or dementia, and age greater than or equal to 55 years. We included research in which at least one of the groups had performed an intervention, either acute (a single session of high-intensity exercise) or chronic (repeated sessions of high-intensity exercise over the course of days, weeks, or months).

### 2.2. Information Sources

Data collection took place from December 2021 to January 2022 using five electronic databases: MEDLINE PubMed, Web of Science, Scopus, CINAHL, and Cochrane plus. A “snowball” search was also conducted to identify additional studies from the reference lists of eligible publications.

### 2.3. Search Strategy

The keywords used were “high intensity functional training”, “intensive functional exercise”, “high intensity functional motor training”, “intensive functional training”, “high intensity functional exercise”, “intermittent exercise”, “circuit training”, “interval exercise”, “intensive functional motor training”, “cognitive impairment”, “dementia”, “older adults”, “older”, “elder”, “elderly”, “older people”, “elderly people”, “aged”, “geriatric” and “senior”. The search was limited by language, publication date, type, and specie. Table 1 shows the search strategies for the different databases.

### 2.4. Selection Process

First, two of the authors discarded duplicate articles as well as those that were clinical records (Y.R.-V., D.F.A.-R.). Different authors independently screened the titles and abstracts of the retrieved articles to exclude items that did not meet the eligibility criteria described above (G.C.V.-Á., P.A.G.-G., A.M.-A., F.L.-M.). Finally, two of the authors independently reviewed the full-text articles for compliance with the inclusion criteria (Y.R.-V., A.A.-A.). Discrepancies were resolved by consensus after consultation with a third author (F.H.-C.). None of the authors of this review were blinded to the journal titles, authors, or institutions featured in the studies.

### 2.5. Data Collection Process

The results of the searches were handled using the Rayyan QCRI application (https://rayyan.qcri.org/welcome, accessed on 29 December 2021). A pre-selection of the studies was made for “title-abstract” according to the inclusion and exclusion criteria described above. The documents selected were later read and synthesized.

### 2.6. Data Items

The present study had as its main outcome the efficacy of HIFT on general cognition and balance as a secondary variable. Any general cognition variable was eligible for inclusion. Results could be reported as an overall test score across multiple domains of general cognition, deriving from tests that provided a specific measure of cognitive function (such as verbal fluency), or both. The studies selected could use a variety of instruments to measure the same or a similar outcome, for example, reporting measures of general cognition using both the Mini-Mental State Examination and the ADAS-Cog.

Data extracted included: author, year of publication, country, study design, characteristics of trial participants (age, level of cognitive impairment, sample size, and group distribution); intervention (including type, intensity, duration, and frequency); outcomes and measurement tools; measurement time points; unintended effects; attrition; and main findings.

### 2.7. Assessment of Methodological Quality

Two independent authors (D.F.A.-R., A.A.-A.) evaluated the methodological quality and risk of bias of the articles included in this review using the PEDro scale [30]. A third author was consulted when discrepancies arose (F.H.-C.). This scale consists of an 11-item checklist, 8 items assess the risk of bias and two items assess the completeness of the statistical report [31]. This instrument has a maximum score of 10 points, as the first item (“eligibility criteria”) is not used in the final score calculation. Each item can be answered as either “Yes” (1 point) or “No” (0 points). An article is considered to be of “Poor” quality when it scores between 0 and 3, “Fair” when the score is 4–5, “Good” when 6–8, and “Excellent” when >9. The Pedro scale appears to be one of the most promising tools for assessing the methodological quality of physical therapy trials [32]

## 3. Results

### 3.1. Selection of the Studies

**Figure 1:**
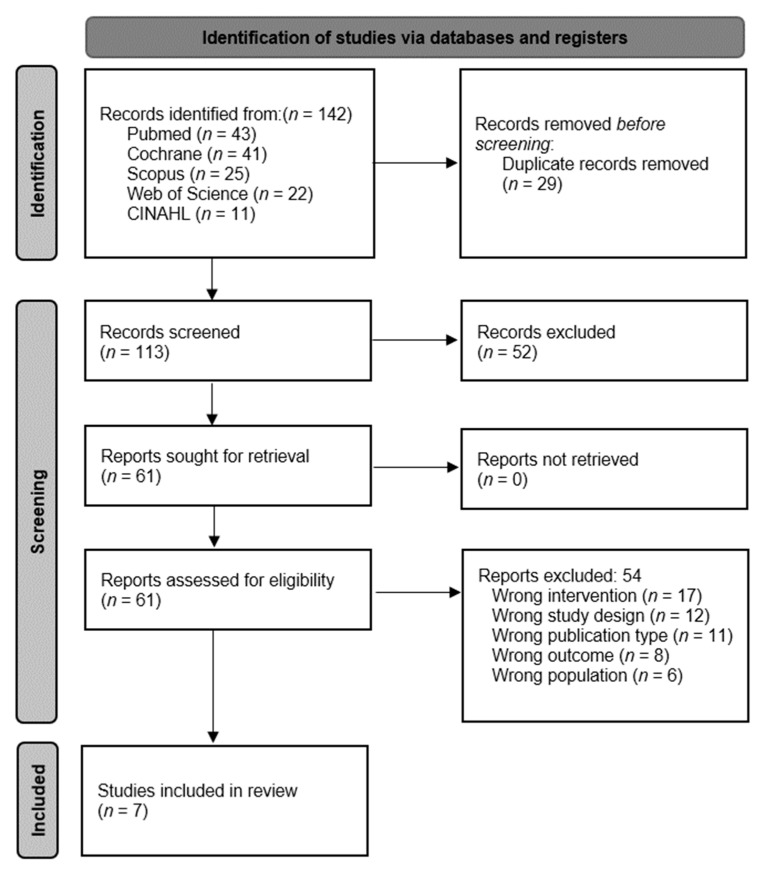
Flow diagram of the study selection process.

A full search was carried out in different databases, which resulted in a total of 142 articles. Then, a filtering of duplicate articles was performed, leaving 113 unique articles. The titles and abstracts of these 113 articles were then reviewed, which left 61 articles as potentially eligible. Only 7 articles met the inclusion criteria [33,34,35,36,37,38,39], as the other 54 were excluded (Figure 1). 1 article [40] appeared to meet the inclusion criteria judging by its title and abstract. However, upon closer reading, the article was excluded because its results were not related to general cognition.

### 3.2. Methodological Quality

Methodological quality was assessed using the PEDro scale. The scores for 6 of the articles were calculated on the PEDro website [33,37,39] while one article [38] was assessed manually. All of the articles included in this review scored between 6 to 8 points, which puts them in the “good quality” category. The mean PEDro score was 7.55 ± 0.72 pt. None of the articles selected blinded the therapists or the assessors [33,34,35,36,37,38,39] one article did not present a between-groups comparison [33], and one article had no similar groups at baseline and did not conceal the allocation [34]. Table 2 presents the results of the PEDro assessment.

### 3.3. Characteristics of the Studies

The articles included in the systematic review were randomized controlled trials published in Sweden [36,39], Norway [37,38], UK [35], Nigeria [34], and Australia [33] during 2011 and 2020 (2011 [36], 2014 [33], 2015 [37,38], 2017 [39], 2018 [35], and 2020 [34].

A total of 1152 persons (52.30% women and 47.70% men) participated in the studies analyzed. Their age was 76.9 on average (±11.4), ranging from 55 to 100 years, which is in accordance with the inclusion criteria. People with mild or moderate dementia (MMSE ≥ 10) [33,34,35,36,37,38,39] were included. A total of 764 individuals were assigned to the various experimental groups with HIFT protocols [33,34,35,36,37,38,39], 462 to the control groups [33,34,35,36,37,38,39], and 22 people were part of other, non HIFT experimental groups [33] (Table 3).

### 3.4. Outcomes

The main variable studied was general cognition and the effects that different HIFT programs have on it. One article used the Mini-Mental State Examination to assess general cognition [36], three articles used a combination of MMSE and the Alzheimer’s Disease Assessment Scale-cognitive (ADAS-Cog) [34,35,39], two articles used MMSE and the Clinical Dementia Rating Scale (CDR) [37,38], and finally, one article assessed general cognition with the ADAS-Cog only [33].

As additional data, the studies authored by Toots et al. [41], Littbrand et al. [36], and both pieces by Telenius et al. [37,38] measured balance using the Berg Scale (BBS).

### 3.5. Study Intervention

Every study [33,34,35,36,37,38,39] included at least one experimental group with a HIFT program intervention. Four studies [36,37,38,39] focused the HIFT intervention on the lower limbs and combined strength and balance exercises. One article [35] used a HIFT program based on aerobic and strength exercises. Two articles [33,34] used a progressive HIFT program. Fiatarone et al. [33] based progression on the judgment of the researcher guided by a daily assessment of the Borg Scale, always aiming at a 15–18 score. They also started at 80% of the RM and every 3 weeks a 3% adjustment was made to maintain high intensity. As for Gbiri et al. [34], to set the intensity of the training the researchers determined the maximum repetition and/or pace for each exercise: participants started at 80% of maximum effort, progressing by 10% per week for two weeks. Once the participant adapted to the intensity, the process was repeated.

To ensure that a high level of intensity was maintained, the researchers used a variety of methods. One study [33] used a combination of the Borg Scale and a percentage of RM. Lamb et al. [35] used the 6-min walk test to measure intensity as per the indications of Luxton et al. [42], tailored it to the fitness and health status of the participant after an individual assessment that considered conditions such as pathologies and treatments. One study [34] assessed maximum repetition and pace for each individual and then had them work at 80% of that intensity. Finally, four studies [36,37,38,39] set the intensity at 12 repetitions with maximum load combined with a high-intensity self-paced rhythm.

The intervention duration varied between 12-week [34,37,38], 13-week [36], 16-week [35,36,37,38,39] and 26-week [33] protocols. All studies set a frequency of two sessions per week [33,34,35,36,37,38,39].

One study [33] presented other experimental groups than HIFT. Despite their focus on physical activity, they did not meet the inclusion criteria of being functional, so they were not considered for final inclusion.

As for the control groups, interventions ranged from exercises led by occupational therapists [36], singing and listening to music [37,38,39], light physical activity while sitting [37,38], basic at-home exercise programs [34], or a sham cognitive and physical exercise protocol [33]. It should be noted that none of the articles reported any objective assessment of intensity in the control group activities [33,34,35,36,37,38,39].

### 3.6. Study Results

Balance was assessed in three of the articles [36,37,38], which employed the Berg scale. Two of those [37,38] showed that a HIFT intervention has long-term positive effects on balance, while the other [36] failed to find a correlation between HIFT and balance.

Two articles [33,34] showed that a progressive HIFT program improves general cognition. However, one article [35] concluded that a HIFT intervention does not slow cognitive decline and might in fact worsen it. Finally, four articles [36,37,38,39] showed no significant changes within or between groups. 

## 4. Discussion

This systematic review, which was carried out to determine the effects of HIFT on the cognitive performance of cognitively impaired older adults, considered seven articles that met the inclusion criteria [33,34,35,36,37,38,39]. After reviewing these studies, no consensus was reached concerning the effects of HIFT on the cognitive function of the individuals under assessment. This disparity regarding the primary outcome may be attributed to several methodological differences between the studies.

One of the main methodological differences found in this review was related to the instruments used to assess changes in general cognition. In the studies included, changes in general cognition were assessed using ADAS-cog, MMSE, or a combination of both, as detailed in the results. However, despite this discrepancy similar conclusions could be reached regarding the burden of disease using either of these two instruments. As a matter of fact, Khandker et al. [43], evaluated the comparability of ADAS-cog and MMSE, finding a significant association between MMSE and ADAS-cog (*p* < 0.001, R2 = 0.561, in 813 patients and 1520 MMSE/ADAS-cog paired measurements) where increases by 2.01 points (95% CI [1.90, 2.11]) of ADAS-cog were associated with decreases by one point for MMSE. These results were consistent with those reported later by Levine et al. [44].

Furthermore, we identified variability in the HIFT protocols, which was expected because this training modality uses constantly varied, multi-joint exercises of varying duration, with or without rest periods [14]. All the articles included resistance training in their respective HIFT protocols, but we found differences in the methodologies used to prescribe the training load. Two articles [34,37] used intensity-based prescription (%1 RM), while the remaining five articles [35,36,38,39] used a volume measure (the number of repetitions). Despite these two measures usually being correlated, recent research has raised doubts about the accuracy of this correlation [45]. It has been reported that the amount of muscle mass used during exercise influences the number of repetitions performed at a given percentage of 1 RM [46]. Likewise, intensity (expressed as %1 RM) and volume (expressed as the number of repetitions), when used as the only measures of training load control, are insufficient to correctly prescribe this type of training, as it is necessary to control variables such as inter-set recovery duration [47], the predominance of the eccentric or concentric phase [48], and speed of execution [49]. These variations influence force production and other hormonal [49] and neuromuscular responses [50]. In addition, there is evidence for a positive association between movement speed and cognition in older adults [51], and it has been reported that a greater cognitive load is required in eccentric-predominant exercises compared to concentric-predominant ones [52]. On the other hand, some differences found in the load progression strategies should be pointed out, which could induce different adaptations with respect to load volume [53]. In the strategy used by Gbiri et al. [34] the rate of execution of the exercises was monitored, increasing by 10% every 2 weeks. Additionally, the same authors reported an initial measure of the load equal to 80% RM, with no progressions in this regard. In contrast, Fiatarone et al. [33] adjusted the initial intensity to 80% RM and performed progressions of 3% every 3 weeks. Meanwhile, in five of the articles reviewed [35,36,37,38,39] a measure of up to 12 RM was used for intensity control, with increases in load once the participant was able to easily perform more than 12 repetitions.

On the other hand, although the benefits of exercise on cognitive function are well documented [54,55,56], a recent review and meta-analysis revealed that there was no beneficial effect of exclusively-HIIT-based interventions on the cognitive functioning of people with dementia [57]. In contrast, it has been reported that programs based on functional exercise may have certain positive effects on cognitive function in older adults with mild cognitive impairment (MCI) [58]. Furthermore, HIFT has been administered to older adults with moderate to severe dementia in nursing homes, generating in this population joy and rediscovery of body competencies, as well as a secure adherence to the activities and understanding of the objectives of the exercises [59]. Likewise, the applicability of such interventions has been successfully evaluated in relation to the exercise intensity achieved [60]. However, the results of this review on the effect of HIFT on general cognition are varied and in some cases contradictory. One of the studies included [33], which evaluated older adults with MCI, showed that six months of HIFT-based resistance training doubled the proportion of participants with normal ADAS-Cog scores. Additionally, other analyses on this same population, in a different arm of the same study (The Study of Mental and Resistance Training—SMART), showed that the existence of initial cognitive impairment does not prevent the development of physical adaptations, and that improvements in muscle strength are related to cognitive adaptations [61]. Similarly, we found that both six [34] and twelve [35] months of HIFT were able to induce improvements in global cognition among older adults with dementia. This, however, was in contrast with four other studies [36,37,38,39] in which the HIFT-based intervention did not improve global cognition in adults with mild or moderate dementia.

In addition, this review analyzed the impact of HIFT on balance, which was assessed in three of the studies included [36,37,38]. Judging by the results, it is clear that a 12-week HIFT intervention was able to improve balance in adults with dementia (mild and moderate) [36,37,38]. This notwithstanding, it is remarkable that no combined improvement in balance and global cognition was reported in response to the HIFT intervention. This could be explained by the low sensitivity of the MMSE to identify executive dysfunction compared to other tests such as the Montreal Cognitive Assessment (MoCA) [62,63]. This is particularly relevant considering that balance control has been reported to deteriorate as the severity of cognitive impairment increases, and that executive function is essential for balance control [64]. This last observation is consistent with a study whose results confirm that combined training (motor and cognitive) improves frontal cognition (assessed with MoCA) and balance in people with Alzheimer’s disease [65]. Similar results are also observable in middle-aged and older adults without dementia, and it was recently reported that improved global cognitive function, also assessed with MoCA, could be associated with improvements in balance after a 12-week exercise intervention (Tai Chi). In the latter case, such association could be linked to improvements in lower-limb strength [66].

This systematic review has several strengths such as having studied with good methodological quality (internal validity), including a population with a wide age range (55–100 years), and being homogeneous in relation to sex (similar number of men and women). However, some limitations were identified including the fact that the great methodological heterogeneity in the protocols implemented limits the possibility of deriving concrete results of HIFT on general cognition in older adults with dementia. Therefore, it is necessary to design interventions with greater methodological rigor that lead to an adequate understanding of the effects of exercise on the cognitive changes that accompany aging. Moreover, the degree of statistical heterogeneity of the articles included in this review was not assessed, which limits the possibility of measuring to what extent the results of the different studies can be summarized in a single measure. Additionally, it is possible to identify a geographic bias since the articles included are from Europe (71.4%), Africa (14.28%), and Australia (14.28%), without including research conducted in Asia or America, possibly limiting the generalizability of the results of this review.

## 5. Conclusions

Evidence exists of the benefits of HIFT on general cognition in older adults with cognitive impairment, assessed using the MMSE, the ADAS-cog, or both. Two articles that showed improvement in cognitive function used progressive HIFT with 80% RM at 6, 12, and 18 weeks; on the other hand, studies with HIFT interventions at intensities of 12 RM find no significant differences at 3, 4, 6, 7 or at 12 months. However, due to the heterogeneity of intervention protocols, measurement time points, and control group activities, divergent results were evidenced. It is still necessary to determine the modality (load and duration) that guarantees the effectiveness of the intervention.

It is important to emphasize that more studies are still needed that conduct a better follow-up of the activities in the control group, as well as the standardization of an instrument used to assess general cognition and a more rigorous design of the intervention. Such design should consider, for instance, the speed of exercise execution, the type of contraction (concentric or eccentric), and the inter-set recovery period. Only thus could it be possible to ascertain with precision the possible effects induced by the intervention and their duration over time. The unification of concepts is required both in the intervention and the measurement of the variables in the RCTs in order to elucidate the effects of HIFT on general cognition in older adults with mild to moderate cognitive impairment.

## Figures and Tables

**Table 1:** Search strategy.

Databases	Search Strategy	Limits	Filter
MEDLINE Pubmed	(“high intensity functional training” OR “intensive functional exercise” OR “high intensity functional motor training” OR “intensive functional training” OR “high intensity functional exercise” OR “intermittent exercise” OR “circuit training” OR “interval exercise” OR “intensive functional motor training” OR “HIFT”) AND (“cognitive impairment” OR “dementia”) AND (“older adults” OR “older” OR “elder” OR “elderly” OR “older people” OR “elderly people” OR “aged” OR “geriatric” OR “senior”)	Published date: 2011–2021; Clinical study	43
Cochrane		Published date: 2011–2021; Trial	41
Scopus		Published date: 2011–2021; Article; Humans.	25
Web of Science		Published date: 2011–2021; Articles	22
CINAHL		Published date: 2011–2021; Randomized controlled trial	11

**Table 2:** Risk of bias and methodological quality of the articles included.

Items Authorship	1	2	3	4	5	6	7	8	9	10	11	Total
Fiatarone et al., 2014 [33]	Y	Y	Y	Y	N	N	Y	Y	N	Y	Y	7
Lamb, et al., 2018 [35]	Y	Y	Y	Y	N	N	Y	Y	Y	Y	Y	8
Littbrand, et al., 2011 [36]	Y	Y	Y	Y	N	N	Y	Y	Y	Y	Y	8
Gbiri et al., 2020 [34]	Y	Y	N	Y	N	N	Y	N	Y	Y	Y	6
Telenius, et al., 2015 [37]	Y	Y	Y	Y	N	N	Y	Y	Y	Y	Y	8
Telenius, et al., 2015 [38]	Y	Y	Y	Y	N	N	Y	Y	Y	Y	Y	8
Toots et al., 2017 [39]	Y	Y	Y	Y	N	N	Y	Y	Y	Y	Y	8

Items: 1 = eligibility criteria; 2 = random allocation; 3 = concealed allocation; 4 = baseline comparability; 5 = blind subjects; 6 = blind therapists; 7 = blind assessors; 8 = adequate follow-up; 9 = intention-to-treat analysis; 10 = between-group comparisons; 11 = point estimates and variability; Y = Yes; N = No.

**Table 3:** Characteristics of the included studies.

Author	Sample (I/C)	Age	Intervention	Intensity	Control	Measuring Instrument	Assessments	Results
Fiatarone et al., 2014 [33]	73/27	55–89	EG1: CT and Progressive HIFT. EG2: HIFT and SCOG. EG3: CT and SPEX	15–18 on the Borg Scale and 80% RM	SCOG and SPEX	ADAS-Cog	T0 = Baseline T1 = 6 months T2 = 18 months	6 months intervention of a HIFT program improves global cognition compared to sham exercise (*p* < 0.05); this benefit persisted for 18 months (*p* = 0.08).
Lamb, et al., 2018 [35]	329/165	77 ± 7.9	HIFT	RPE adapted for use by people with dementia 20 RM 12 RM	Usual Physical Activity of the Participant.	MMSE ADAS-Cog	T0 = Baseline T1 = 6 months T2 = 12 months	4 months intervention of HIFT that includes aerobic and strength exercise has negative effects on the cognitive impairment in people with mild to moderate dementia (adjusted mean difference −0.6; 95% confidence interval −1.6 to 0.4; *p* = 0.24). Cognitive impairment declined over the 12-month follow-up in both trial arms; (adjusted mean difference −1.4, 95% confidence interval −2.6 to −0.2) *p* = 0.03.
Littbrand, et al., 2011 [36]	91/100	85.3 ± 6.1	HIFT	8–12 RM	Occupational Therapist Exercise Program developed exclusively for this study.	MMSE Berg Scale	T0 = Baseline T1 = 3 months T2 = 6 months	No significant differences were found between the groups after the intervention. After 3 months *p* = 5.28 and after 6 months *p* = 0.47.
Gbiri et al., 2020 [34]	16/15	69.6 ± 3.4	Progressive HIFT	80% RM	Basic Home Exercise Program.	MMSE ADAS-Cog	T0 = Baseline T1 = 6 weeks T2 = 12 weeks	Progressive HIFT improves cognitive function. MMSE (mean rank) between baseline and post 6-week interventions: 3.56 for experimental group and 1.20 for control group- *p* = 0.022. MMSE between 6-week and 12-week of intervention 3.75 (experimental group), 1.87 (control group) *p* = 0.000.
Telenius, et al., 2015 [37]	87/83	86.7 ± 7.4	HIFT	12 RM	Light physical activity in sitting.	MMSE CDR Berg Scale	T0 = Baseline T1 = 12 weeks	No significant changes in the MMSE (*p* = 0.69, effect size 0.1)
Telenius, et al., 2015 [38]	87/81	86.9 ±7.4	HIFT	12 RM	Light physical activity, reading, playing games, listening to music and conversations	MMSE CDR Berg Scale	T0 = Baseline T1 = 3 months T2 = 6 months	Post-intervention measures showed no significant differences between groups *p* = 0.492.
Toots et al., 2017 [39]	93/93	85.1 ± 7.1	HIFT	8–12 RM	While seated they sang, listened to music or readings, and/or looked at pictures and objects concerning interesting topics.	MMSE VF ADAS-Cog	T0 = Baseline T1 = 4 months T2 = 7 months	There were no differences from baseline between groups at 4 months (−0.27, 95% CI −1.4 to 0.87, *p* = 0.644) or at 7 months in MMSE (−1.15, 95% CI −2.32 to 0.03, *p* = 0.056)

N: number of participants. CG: control group. EG: experimental group. HIFT: High-Intensity Functional Training. CT: cognitive training. SCOG: sham cognitive. SPEX: sham physical. MMSE: Mini-Mental State Examination. ADAS-Cog: Alzheimer’s Disease Assessment Scale-cognitive. CDR: Clinical Dementia Rating Scale. 6 MWT: 6-Minute Walk Test. RM: repetition maximum. T: measurement time points.

## Data Availability

All available data can be obtained by contacting the corresponding author.
